# Self-reported sleepiness and not the apnoea hypopnoea index is the best predictor of sleepiness-related accidents in obstructive sleep apnoea

**DOI:** 10.1038/s41598-020-72430-8

**Published:** 2020-10-01

**Authors:** P. Philip, S. Bailly, M. Benmerad, J. A. Micoulaud-Franchi, Y. Grillet, M. Sapène, I. Jullian-Desayes, M. Joyeux-Faure, R. Tamisier, J. L. Pépin

**Affiliations:** 1 SANPSY-USR 3413, SANPSY-CNRS, Bordeaux University, 33000 Bordeaux, France; 2 HP2 Laboratory, INSERM U1042, Grenoble Alpes University, Grenoble, France; 3 EFCR Laboratory, Grenoble Alps University Hospital, Pole Thorax et Vaisseaux, Grenoble, France; 4 Private Practice Sleep and Respiratory Disease Centre, Valence, France; 5 Private Practice Sleep and Respiratory Disease Centre, Nouvelle Clinique Bel Air, Bordeaux, France

**Keywords:** Diagnostic markers, Respiratory tract diseases

## Abstract

To evaluate the value of apnoea + hypopnoea index versus self-reported sleepiness at the wheel in anticipating the risk of sleepiness-related accidents in patients referred for obstructive sleep apnoea. A cross-sectional analysis of the French national obstructive sleep apnoea registry. 58,815 subjects referred for a suspicion of obstructive sleep apnoea were investigated by specific items addressing sleepiness at the wheel and sleepiness-related accidents. Apnoea + hypopnoea index was evaluated with a respiratory polygraphy or full polysomnography. Subjects had a median age of 55.6 years [45.3; 64.6], 65% were men, with a median apnoea + hypopnoea index of 22 [8; 39] events/h. Median Epworth sleepiness scale score was 9 [6; 13], 35% of the patients reported sleepiness at the wheel (n = 20,310), 8% (n = 4,588) reported a near-miss accident and 2% (n = 1,313) reported a sleepiness-related accident. Patients reporting sleepiness at the wheel whatever their obstructive sleep apnoea status and severity exhibited a tenfold higher risk of sleepiness-related accidents. In multivariate analysis, other predictors for sleepiness-related accidents were: male gender, ESS, history of previous near-miss accidents, restless leg syndrome/periodic leg movements, complaints of memory dysfunction and nocturnal sweating. Sleep apnoea per se was not an independent contributor. Self-reported sleepiness at the wheel is a better predictor of sleepiness-related traffic accidents than apnoea + hypopnoea index.

## Introduction

Sleep disorders are a leading cause of traffic accidents^1^ and are associated with more severe injuries and a higher rate of accident-related mortality. Assessment of accident risk can be schematically stratified by identifying typical medical conditions associated with sleepiness and behavioural or lifestyle factors increasing the risk. Estimating fitness-to-drive involves recognizing potential sleep disorders and inappropriate sleep hygiene^2^. Moreover, the studies by Connor et al.^3^ Masa et al.^4^, and Lloberes et al.^5^ showed that self-perceived sleepiness at the wheel before the crash was significantly associated with accident risk. Thus, we believe that physicians should adopt a structured approach for interviews focusing on symptoms and context, sleep habits and inappropriate behaviours. When the clinical context is evocative, sleep studies should also be scheduled.

The European Union has issued recommendations for fitness to drive^6^ in specific diseases (epilepsy, diabetes, obstructive sleep apnoea) but the criteria for risk stratification and appropriate selection for sleep studies are still debated. Obstructive sleep apnoea is a common sleep disorder that can be defined as the repetitive occurrence of complete (apnoea) or incomplete (hypopnoea) pharyngeal collapse during sleep ended by micro arousals. As a result of intermittent hypoxia and sleep fragmentation, obstructive sleep apnoea may be associated with excessive daytime sleepiness but this occurs only in approximately half of the patients^7^. This raises the question of the actual risk of traffic accidents in obstructive sleep apnoea patients not complaining of excessive daytime sleepiness. The value of the indices of obstructive sleep apnoea severity such as apnoea hypopnea index as true predictors of risk at the wheel is also questionable. While several studies showed that apnoeic patients present a higher risk of traffic accidents, they did not clearly differentiate apnoea + hypopnoea index score and the presence of nocturnal breathing disorders plus excessive daytime sleepiness. Masa showed for instance that in a large cohort of nonprofessional drivers, apnoea + hypopnoea index alone was not able to discriminate patients with and without accidents^4^. Another striking point is that professional drivers have a huge prevalence of nocturnal breathing disorders (up to 60%,^8^). However, Stoohs^9^ showed in that population that the severity of apnoea + hypopnoea index was not a predictor of accident risk. In addition, Howard^8^ showed that excessive daytime sleepiness is a much stronger predictor of accidents than apnoea + hypopnoea index. More surprisingly in the study by Howard, AHI explained only single-vehicle accidents while excessive daytime sleepiness explained both single- and multiple-vehicle accidents.

The diagnostic value of apnoea + hypopnoea index versus specific symptoms of sleepiness at the wheel needs thus to be evaluated in large unselected real-life obstructive sleep apnoea populations. Several studies have previously investigated driving risk in obstructive sleep apnoea patients. The range of reported accident risk is highly variable between studies, possibly owing to the heterogeneity of the obstructive sleep apnoea populations studied (i.e. with or without excessive daytime sleepiness), different grades of severity and different methodologies to identify and analyse accidents (self-reported versus objective data from departments of motor vehicle accidents). A first meta-analysis^10^ showed that non-commercial drivers with sleep apnoea are at a statistically significant increased risk of involvement in motor vehicle accidents. However, the relationship between risk and daytime sleepiness and/or the sleep apnoea severity was not consistent across the studies. A possible explanation is that sleepiness was mainly assessed by the ESS which investigates various daily life situations including very passive ones such as listening to a speech, lying down and watching TV, which might not represent properly and specifically mirror sleepiness at the wheel. A more recent meta-analysis dedicated to sleepiness at the wheel and driving accidents^11^ reported that self-perception of sleepiness at the wheel is a robust predictor of accident risk, independently of the behavioural aspects or organic cause of excessive daytime sleepiness. That meta-analysis demonstrated much stronger correlations between sleepiness at the wheel than what was reported with excessive daytime sleepiness measured by the Epworth sleepiness scale^10^. Considering the existing literature, we believe it is very important to run studies able to clearly demonstrate the separate weight of apnoea + hypopnoea index, on the one hand, and sleepiness at the wheel, on the other, in order to help physicians to take decisions able to decrease the accident risk of their patients**.**

## Objectives

The Observatoire Sommeil de la Fédération de Pneumologie is collecting data in a prospective cohort of patients suspected of having obstructive sleep apnoea^12^. Each patient is evaluated during a structured interview associated with an electronic medical record and is then assessed by respiratory polygraphy or polysomnography to confirm the diagnosis of obstructive sleep apnoea and treat it. Apart from the classical symptoms and comorbidities characterizing obstructive sleep apnoea (in particular excessive daytime sleepiness), this national registry includes questions concerning sleepiness at the wheel, the occurrence of near-miss accidents and self-reports of sleepiness-related accidents. The main goal of the current study was to quantify the true value of apnoea + hypopnoea index versus sleepiness at the wheel in order to better identify obstructive sleep apnoea patients at risk for sleepiness-related accidents.

## Methods

### Study design and setting: the OSFP national registry

Data from a prospective national cohort (www.osfp.fr) were used to conduct this study. All participants were offered the opportunity to join the OFSP during a first consultation motivated by a suspicion of obstructive sleep apnoea, based on clinical complaints (snoring, sleepiness…). The OSFP registry^12^ is a standardized web-based report administered by the French Federation of Pulmonology. It contains anonymized longitudinal data from patients complaining of sleep disorders who have been investigated by respiratory physicians in private practice, general hospitals and university hospitals. Periodic quality control checks are performed to ensure up-to-standard data-recording. Ethical committee approval for setting up the database was obtained from “Le Comité consultatif sur le traitement de l’information en matière de recherche en santé” (CCTIRS no 09.521) and authorisation from the “Commission Nationale Informatique et Liberté”, the French information technology and personal data protection authority. The OSFP Independent Scientific Advisory Committee approved data use for this study. All methods were performed in accordance with the relevant guidelines. All patients included in the database provided written informed consent.

### Sleep studies

The diagnosis of obstructive sleep apnoea was obtained by full-night polysomnography (PSG) performed at a sleep centre or by polygraphy performed at home. For PSG, sleep stages were scored manually according to the American Academy of Sleep Medicine criteria^9^ The scoring of respiratory events was done according to the AASM rules^14^. For PG, hypopneas were scored only when associated with a 3% desaturation in oxygen. Apnoea + hypopnoea index was defined as the number of apnoeas and hypopneas per hour of sleep (full-night PSG) or per hour of recording.

### Variables and data sources

The following data, collected at the first medical visit, were recorded (see supplementary material): demographic characteristics, scores (ESS, Pichot fatigue scale and Pichot depression scale), subjective sleep duration, obstructive sleep apnoea symptoms, office blood pressure, waist circumference and co-morbidities (cardiovascular, metabolic and respiratory). Environmental risk factors such as smoking and alcohol as well as sedentary lifestyle were also collected. Patients were also investigated via a specific questionnaire exploring sleepiness at the wheel, which was defined as "severe episode interfering with driving skills potentially constraining the patients to stop driving to prevent a sleepiness-related accident". Patients also reported if they had had near-miss accidents and sleepiness-related accidents. The global perception of sleepiness was evaluated by physicians on a time frame relevant to clinical symptoms able to justify the consultation, so a period could last from a few weeks up to one year according to the frequency of symptoms.

### Participants

Patients aged at least 18 years at their first visit and who answered the questions about fitness-to-drive were included in the study. All patients reported a sleep complaint or a suspicion of obstructive sleep apnoea.

### Statistical methods

Quantitative data were expressed by using median and interquartile range and qualitative data were expressed by using numbers and percentage. A simple imputation method was performed to replace missing data: quantitative variables were imputed using the median and qualitative variables using the most frequent modality. The population was stratified according to obstructive sleep apnoea occurrence and severity (apnoea + hypopnoea index thresholds at 10 and 30 events/h, respectively) and presence or absence of sleepiness at the wheel. Variables were compared between groups using a Chi squared test for qualitative variables and a non-parametric Kruskal–Wallis test for quantitative variables. Post-hoc tests with Bonferroni correction for multiple tests were performed if the overall test was significant.

To compare the patients according to sleepiness-related accidents, a description of the variables was performed using Chi squared tests to compare qualitative variables between both groups and a Mann–Whitney test to compare non-parametric quantitative variables.

Finally, to identify risk factors associated with sleepiness-related accidents, a univariate logistic regression was performed. Owing to the high sample size, a p value threshold of 0.05 was used to select variables for the multivariable analysis. Apnoea + hypopnoea index and age were forced into the final model. Log-linearity of quantitative variables was tested and, if necessary, variables were categorized. A stepwise multivariate logistic regression was finally performed to identify independent factors of sleepiness-related accidents. Statistical analyses were performed using SAS v9.4 (SAS Institute Inc., Cary, NC, USA). A p-value < 0.05 was considered as significant.

## Results

### Participants (study flow-chart depicted in Fig. 1) and descriptive data

**Figure 1:**
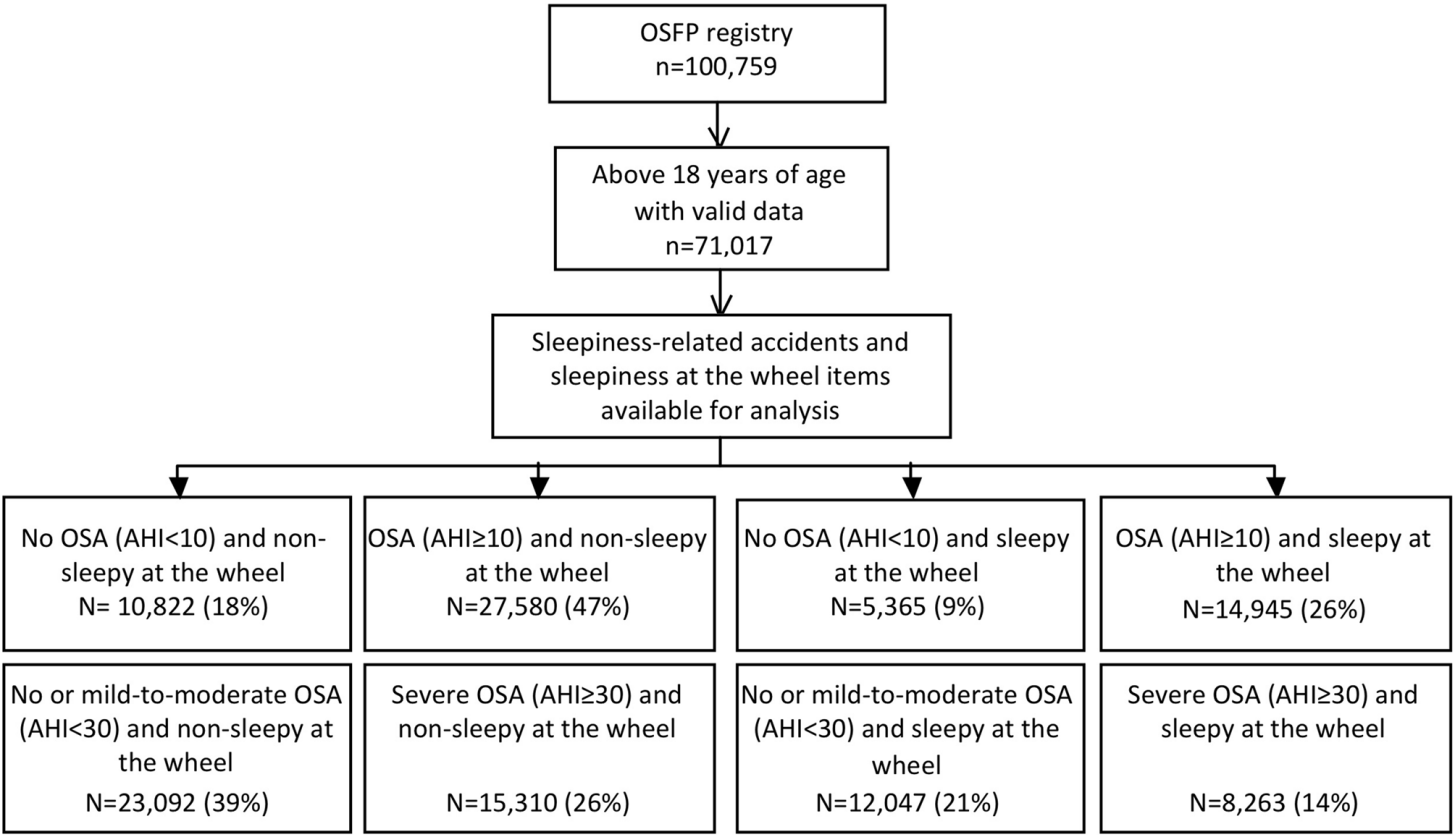
Study flow chart.

Subjects included in the study (n = 58,712) had a median age of 55.6 years [interquartile range: 45.3; 64.6], 65% of them were men, with a median apnoea + hypopnoea index of 22.0 [8.4; 38.8] events/h. The median ESS was 9 [6; 13]. As described in the methods section, the population was stratified according to obstructive sleep apnoea occurrence and severity (apnoea + hypopnoea index AHI thresholds at 10 and 30 respectively) and the presence or absence of self-reported sleepiness at the wheel (Fig. 1). Tables 1 and 2 present the main characteristics of the subgroups. As expected, male gender, higher BMI and age were associated with severe obstructive sleep apnoea. As shown in Tables 1 and 2, imputation did not impact the results.

**Table 1:** Non-OSA and OSA subgroups sleepy or not at the wheel.

	Non-sleepy at the wheel		Sleepy at the wheel		
Variable	Non OSA (AHI < 10) n = 10,822	OSA (AHI ≥ 10) n = 27,580	Non OSA (AHI < 10) n = 5,365	OSA (AHI ≥ 10) n = 14,945	p
Age (years)	50.7 [39.9; 61.4]	59.2 [49.8; 67.7]	46.2 [37.3; 55.6]	54.6 [45.9; 62.6]	< .01
Gender (numbers (% men))	5,599 (51.8)	18,582 (67.6)	2,866 (53.5)	11,048 (74.1)	< .01
Body mass index (kg/m^2^)	28.7 [24.8; 34.3]	30.7 [26.9; 35.5]	27 [23.9; 31.2]	29.9 [26.4; 34.4]	< .01
Apnoea + hypopnoea index—AHI (events/h)	4 [2; 6.9]	31 [19; 45.5]	4.2 [2; 7]	31 [19; 47.8]	< .01
Epworth ≥ 10	3,334 (36.8)	9,445 (40.9)	3,348 (64.1)	9,350 (65.6)	< .01

Comparison were made for all groups (p) and between groups by using post-hoc test with Bonferroni correction (corrected p value threshold: < 0.0083). All comparison remains significant in post-hoc test, excepted between No OSA groups for sex, between OSA groups for apnoea + hypopnoea index (AHI) and between sleepy groups for ESS.

**Table 2:** Non-OSA and mild-to-moderate OSA versus severe OSA subgroups sleepy or not at the wheel.

	Non-sleepy at the wheel		Sleepy at the wheel		
Variable	Non and mild-to-moderate OSA (AHI < 30) n = 23,092	Severe OSA (AHI ≥ 30) n = 15,310	Non and mild-to-moderate OSA (AHI < 30) n = 12,047	Severe OSA (AHI ≥ 30) n = 8,263	p
Age (years)	54.4 [43.6; 64.2]	60.7 [52; 69]	50 [40.5; 58.9]	56.2 [47.9; 63.8]	< .01
Gender (numbers (% men))	13,139 (57)	11,042 (72.3)	7,334 (61)	6,580 (79.8)	< .01
Body mass index (kg/m^2^)	29.1 [25.4; 34.4]	31.5 [27.8; 36.3]	27.8 [24.6; 32]	31.2 [27.7; 35.7]	< .01
Apnoea + hypopnoea index—AHI (events/h)	10.3 [4.2; 18.7]	43 [34; 57]	11 [5; 19]	45 [35; 61]	< .01
Epworth ≥ 10	7,283 (37.3)	5,496 (43.5)	7,459 (63.9)	5,239 (67)	< .01

Comparison were made for all groups (p) and between groups by using post-hoc test with Bonferroni correction (corrected p-value threshold: < 0.0083). All comparisons remain significant in post-hoc test.

### Outcome data

Thirty-five percent of included subjects (n = 20,310) self-reported sleepiness at the wheel, 8% (n = 4,588) reported a previous near-miss accident and 2% (n = 1,313) reported a sleepiness-related accident. Patients reporting sleepiness at the wheel also presented with significantly higher ESS scores versus those reporting no sleepiness at the wheel (13.0 [9.0; 16.5] vs 9 [6; 13] respectively, p < 0.01). Patients reporting a sleepiness-related accident were significantly younger, with a lower BMI and reported more sleepiness at the wheel and a more frequent history of near-miss accidents.

## Main results

*Respective impact of* apnoea + hypopnoea index *and self-reported sleepiness at the wheel on accident risk.*

**Figure 2:**
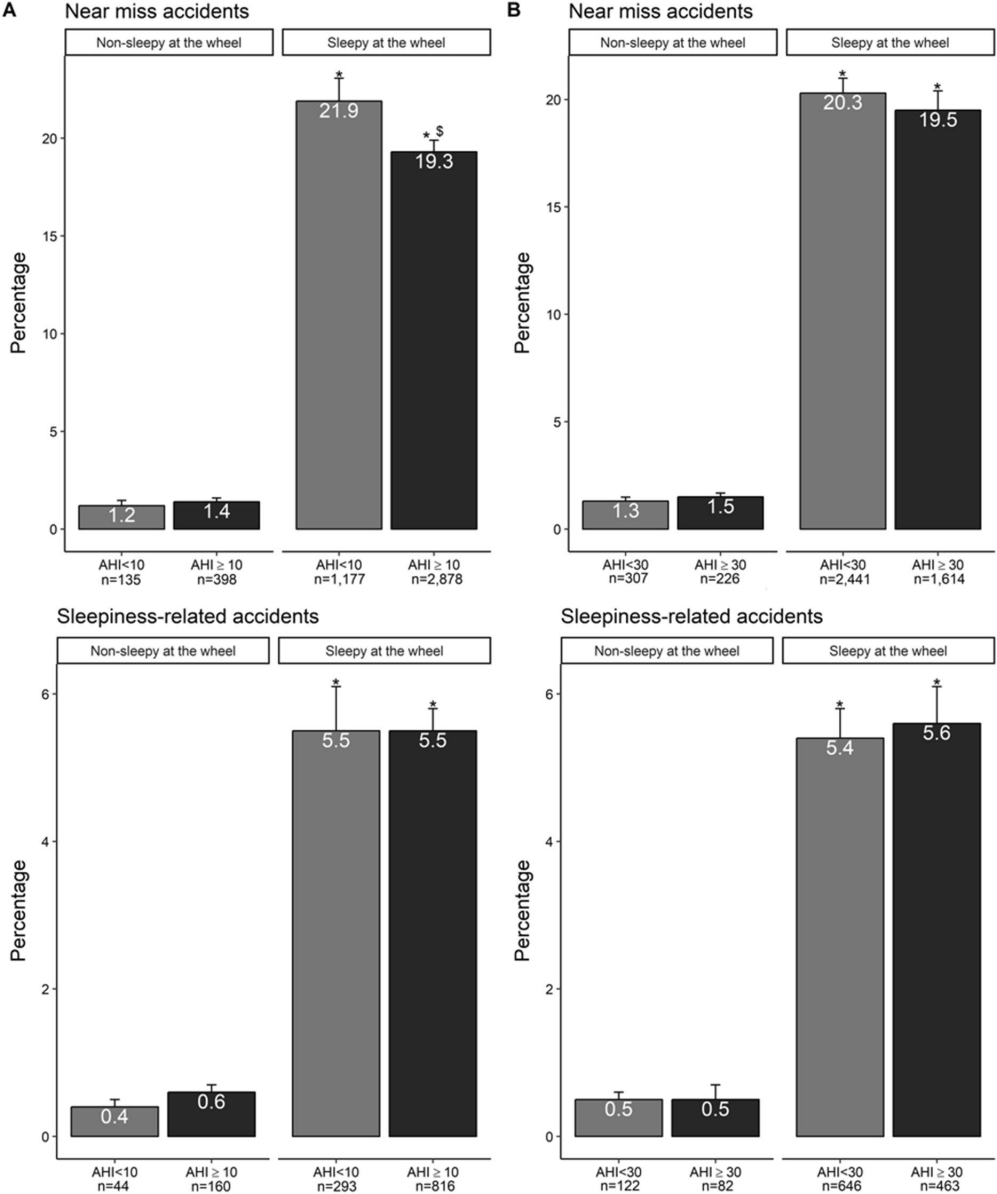
Accident risk in subgroups stratified for OSA severity and sleepy or not at the wheel. Part (**A**): AHI < 10: Non OSA patients; AHI ≥ 10: OSA patients. Part (**B**): AHI < 30: Non and mild-to-moderate OSA patients; AHI ≥ 30: Severe OSA patients. *Significant difference vs. both groups non-sleepy at the wheel (p < 0.01). ^$^significant difference vs. group sleepy at wheel with AHI < 10 (p < 0.01).

Obstructive sleep apnoea patients (with an apnoea + hypopnoea index ≥ 10 or ≥ 30) without sleepiness at the wheel were not at higher accident risk than their respective controls (apnoea + hypopnoea index < 10 or < 30 and no sleepiness at the wheel). Patients reporting sleepiness at the wheel whatever their obstructive sleep apnoea status and severity exhibited a tenfold higher risk of sleepiness-related accidents (p < 0.01) (Fig. 2).

### Multivariate analysis

**Figure 3:**
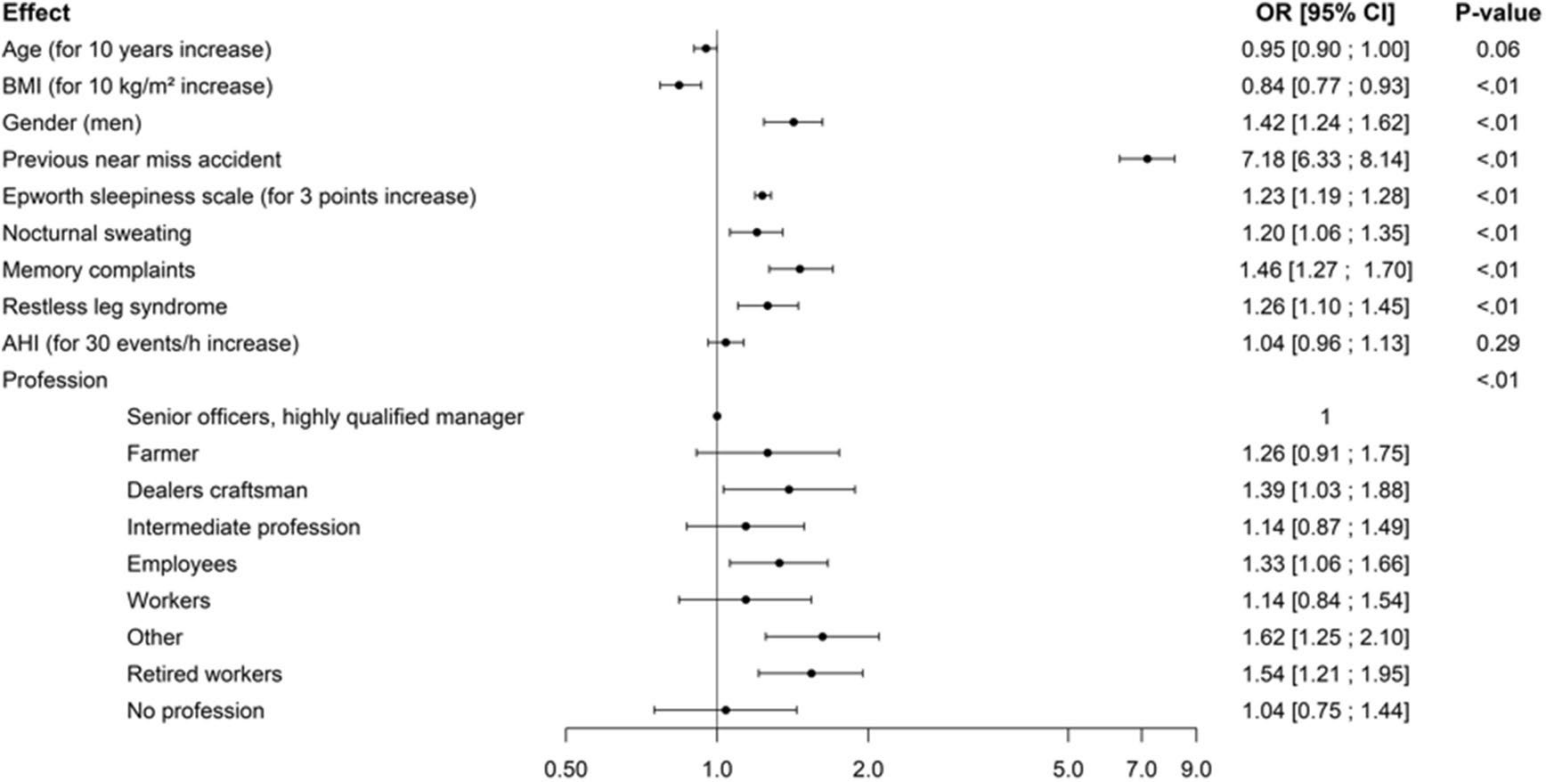
Multivariate analysis.

In multivariate analysis independent predictors of sleepiness-related accidents were: male gender, ESS (each 3-point increase in ESS being associated with a 23% higher risk), history of previous near-miss accidents (sevenfold increased risk), restless leg syndrome/periodic leg movements, complaints of memory dysfunction and nocturnal sweating (Fig. 3). Sleep apnoea per se was not an independent contributor. Concerning BMI, each 10 kg/m^2^ increase in BMI was associated with a 16% lower risk. Concerning professional activity, retirement was associated with a 54% higher risk of sleepiness-related accidents in comparison with senior officers and highly qualified managers.

## Discussion

### Key results

This study in a large sample of patients referred for suspicion of obstructive sleep apnoea (> 50,000) assessed the value of apnoea + hypopnoea index versus self-reported sleepiness at the wheel regarding the risk of sleepiness-related traffic accidents. Because sleepiness at the wheel has a very strong collinearity with sleep-related near misses, we removed sleepiness at the wheel from the regression analysis in order to obtain a better understanding of the main predictive accident factors. Separate analysis using sleepiness at the wheel instead of near misses did not change the predictive value of apnoea + hypopnoea index in the multiple regression analysis model (see supplementary material), and the effect size for sleepiness at the wheel from this model was 9.30 [7.91 ; 10.93]. The studied population truly reflected different sleep clinic practices since it included patients form both private and academic centres, and it was representative of the real-life situation of obstructive sleep apnoea patients. We therefore believe that our results are generalizable.

Previous studies demonstrated that the correlation between indices of obstructive sleep apnoea severity and situational sleepiness as measured by the Epworth sleepiness scale is weak^3,4,15^. We clearly showed that a relatively limited percentage of patients presenting with an obstructive sleep apnoea (apnoea + hypopnoea index AHI ≥ 10) reported sleepiness at the wheel. Previous large epidemiological studies in the general population^16^ also demonstrated that the percentage of subjects with an abnormal apnoea + hypopnoea index (AHI) and who are sleepy is low. This suggests that the severity of excessive daytime sleepiness has measured by the Epworth sleepiness scale associated with a pathological AHI is not the best clinical measure of sleepiness at the wheel and therefore should not be the reference scale to quantify sleepiness in the specific topic of driving risk^17^. Labour-intensive, highly specialised and costly methods for measuring objective sleepiness and the ability to drive should be restricted to a better-defined sub-population following an initial risk stratification based on simple tools available in routine clinical practice.

### Interpretation

Our results demonstrate the validity of self-reported sleepiness at the wheel and history of near misses for anticipating self-reported sleepiness-related traffic accidents. This has previously been reported in several surveys not limited to obstructive sleep apnoea patients and is confirmed here in an unselected obstructive sleep apnoea population^1,18^. By using four different categories (obstructive sleep apnoea (positive/negative) combined with sleepiness at the wheel (positive/negative)), we confirm that an abnormal apnoea + hypopnoea index at a moderate (apnoea + hypopnoea index ≥ 10) or severe level (apnoea + hypopnoea index ≥ 30) is clearly not the best predictor of accident risk**.** Patients should not be considered fit or unfit to drive according to the sole criterion of apnoea + hypopnoea index. This finding has major societal and public health implications because the current interpretation of European regulations, which is possibly insufficiently evidence-based, has led several countries including the UK to dissuade patients with a high apnoea + hypopnoea index from driving a car^19^.

Our multivariate analysis provides additional insights regarding the factors contributing to sleepiness-related accidents in obstructive sleep apnoea. Age and male gender are known risk factors^1^. A higher BMI in our sample was associated with a decreased risk of accidents. This goes against previously published data in professional drivers^9,20^. Driving risk in obese subjects has been explained by an increased level of sleepiness, independently of apnoea + hypopnoea index, possibly related to low-grade inflammation and elevated cytokines^21^. Employees and workers had a higher risk of accidents than senior officers and highly qualified managers, which confirms the increased driving risk of low-income professions. Nocturnal sweating is a marker of high nocturnal sympathetic activity and might represent associated comorbidities and poor sleep quality. The role of restless leg syndrome and periodic leg movements is important and the association of obstructive sleep apnoea with PLMs/poor sleep has been recently shown to be a deadly association for cardiovascular diseases^22,23^. Our data complement this observation by showing that this OSA-PLMs phenotype is also at high risk of sleepiness-related accidents, a finding that should be considered for the purpose of risk stratification in clinical practice^24^. Finally, memory complaints were independently associated with accident risk, as previously reported in studies looking at accident risk in patients with cognitive impairment^25^. Altogether, the data obtained in this large sample reveal a clearer picture of the typical obstructive sleep apnoea patient at high accident risk: a non-obese male with low income, self-reporting sleepiness at the wheel, and with an accrued risk if restless leg syndrome/PLMs are associated. To reduce the burden of the consumption of obstructive sleep apnoea -related resources, there is a need for personalized care and decisions to be made together with patients by using information documenting the simple predictors used in the present study. This finding now needs to be corroborated by prospective external validation studies.

### Limitations

Our study has several limitations. First, we did not collect duration of driving on a yearly basis so we cannot investigate the influence of driving exposure on either traffic accidents or frequency of sleepiness at the wheel. Second, sleepiness at the wheel and sleep-related accidents were collected retroactively, which may introduce a bias in data collection. Even if many other studies on accidents have used similar techniques, there is a need for new prospective studies in order to confirm data obtained until now by studies on accidents due to sleepiness at the wheel. Third, sleepiness at the wheel and sleep-related accidents were collected by self-reports from the patients, so some accidents may have been omitted. No specific time frame was specified during the clinical evaluation, assuming that the symptoms collected reflected the behavioural disability of the patients. However, at baseline evaluation in the sleep clinic, reasons for underreporting accidents are rather limited compared to a context of fitness-to-drive evaluations related to driving licenses clinical consultations. We believe that our findings on the poor predictive value of the apnoea + hypopnoea index are strong enough to impact practice. The rationale of only using the apnoea + hypopnoea index to determine fitness to drive in obstructive sleep apnoea patients is questioned by our data. In the subpopulation of obstructive sleep apnoea with at-risk phenotypical traits arising from our findings, using an objective measure of the ability to remain awake, such as the maintenance of wakefulness test, to complete the assessment of sleepiness-related accident risk is certainly valid and cost-effective.

## Conclusion

Simple tools for stratifying the risk of sleepiness at the wheel to reduce sleepiness-related accidents need to be developed and validated. Future prospective studies are needed to extend our understanding of sleepiness at the wheel and its contribution to accident risk. However, we believe that the apnoea + hypopnoea index is not the best objective marker for evaluating fitness-to-drive in patients suffering from obstructive sleep apnoea and that physicians should focus more on self-reported sleepiness at the wheel, anthropometrics, socioeconomic traits, and associated sleep disorders.

## Supplementary information

Supplementary information.

## References

[1] Philip P (2010). Sleep disorders and accidental risk in a large group of regular registered highway drivers. Sleep Med..

[2] Philip P, Akerstedt T (2006). Transport and industrial safety, how are they affected by sleepiness and sleep restriction?. Sleep Med. Rev..

[3] Connor J (2002). Driver sleepiness and risk of serious injury to car occupants: population based case control study. BMJ.

[4] Masa JF, Rubio M, Findley LJ (2000). Habitually sleepy drivers have a high frequency of automobile crashes associated with respiratory disorders during sleep. Am. J. Respir. Crit. Care Med..

[5] Lloberes P, Levy G, Descals C, Sampol G, Roca A, Sagales T (2000). Self-reported sleepiness while driving as a risk factor for traffic accidents in patients with obstructive sleep apnoea syndrome and in non-apnoeic snorers. Respir. Med..

[6] Krieger J (2002). Public health and medicolegal implications of sleep apnoea. Eur. Respir. J..

[7] Lévy P (2015). Obstructive sleep apnoea syndrome. Nat. Rev. Dis. Primer.

[8] Howard ME, Desai AV, Grunstein RR, Hukins C, Armstrong JG, Joffe D (2004). Sleepiness, sleep-disordered breathing, and accident risk factors in commercial vehicle drivers. Am. J. Respir. Crit. Care Med..

[9] Stoohs RA, Guilleminault C, Itoi A, Dement WC (1994). Traffic accidents in commercial long-haul truck drivers: the influence of sleep-disordered breathing and obesity. Sleep.

[10] Ellen RLB (2006). Systematic review of motor vehicle crash risk in persons with sleep apnea. J. Clin. Sleep Med. JCSM Off. Publ. Am. Acad. Sleep Med..

[11] Bioulac S (2018). Risk of motor vehicle accidents related to sleepiness at the wheel: a systematic review and meta-analysis. Sleep.

[12] Bailly S (2016). Obstructive sleep apnea: a cluster analysis at time of diagnosis. PLoS ONE.

[13] AASM Scoring Manual Version 2.2 - scoring-manual-preface.pdf [Internet]. [cited 2020 Feb 20]. Available from: https://aasm.org/resources/pdf/scoring-manual-preface.pdf

[14] Berry RB, Budhiraja R, Gottlieb DJ, Gozal D, Iber C, Kapur VK (2012). Rules for scoring respiratory events in sleep: update of the 2007 AASM Manual for the Scoring of Sleep and Associated Events. Deliberations of the Sleep Apnea Definitions Task Force of the American Academy of Sleep Medicine. J. Clin. Sleep Med. JCSM Off. Publ. Am. Acad. Sleep Med..

[15] Terán-Santos J, Jiménez-Gómez A, Cordero-Guevara J (1999). The association between sleep apnea and the risk of traffic accidents Cooperative Group Burgos-Santander. N. Engl. J. Med..

[16] Heinzer R (2015). Prevalence of sleep-disordered breathing in the general population: the HypnoLaus study. Lancet Respir. Med..

[17] Driving | Swiss Society for Sleep Research, Sleep Medicine and Chronobiology (SSSSC). https://swiss-sleep.ch/driving/. 10.4414/smw.2015.w30261 36401527

[18] Powell NB (2007). Sleepy driver near-misses may predict accident risks. Sleep.

[19] Ghosh D, Mackay TW, Riha RL (2016). European Union directive 2014/85/EU on driver licensing in obstructive sleep apnoea: early experiences with its application in the UK. Breathe Sheff. Engl..

[20] Anderson JE (2012). Obesity is associated with the future risk of heavy truck crashes among newly recruited commercial drivers. Accid. Anal. Prev..

[21] Vgontzas AN (2008). Does obesity play a major role in the pathogenesis of sleep apnoea and its associated manifestations via inflammation, visceral adiposity, and insulin resistance?. Arch. Physiol. Biochem..

[22] Zinchuk AV (2018). Polysomnographic phenotypes and their cardiovascular implications in obstructive sleep apnoea. Thorax.

[23] Pépin JL, Bailly S, Tamisier R (2018). Incorporating polysomnography into obstructive sleep apnoea phenotyping: moving towards personalised medicine for OSA. Thorax.

[24] Philip P (2014). Complaints of poor sleep and risk of traffic accidents: a population-based case-control study. PLoS ONE.

[25] Lafont S, Laumon B, Helmer C, Dartigues J-F, Fabrigoule C (2008). Driving cessation and self-reported car crashes in older drivers: the impact of cognitive impairment and dementia in a population-based study. J. Geriatr. Psychiatry Neurol..

