# Supplementary material for: Self-reported sleepiness and not the apnoea hypopnoea index is the best predictor of sleepiness-related accidents in obstructive sleep apnoea
Source: Sci Rep. 2020 Oct 1;10:16267. doi: 10.1038/s41598-020-72430-8 (PMC7529742; doi:10.1038/s41598-020-72430-8)
Supplement: Supplementary file 1 — Supplementary information. [file 41598_2020_72430_MOESM1_ESM.pdf]

Self-reported sleepiness and not the apnoea hypopnoea index is the best predictor of sleepiness-related accidents in obstructive sleep apnoea

P Philip[1*], S Bailly[2,3*], M Benmerad[2,3], JA Micoulaud-Franchi[1], Y Grillet[4], M Sapène[5], I Jullian-Desayes[2,3], M Joyeux-Faure[2,3], R Tamisier[2,3$], JL Pépin[2,3$]

[1]SANPSY-USR 3413, SANPSY-CNRS, FRANCE,

[2]HP2 laboratory, INSERM U1042, Grenoble Alpes University, Grenoble, France

[3]EFCR Laboratory, Pole Thorax et Vaisseaux, Grenoble Alps University Hospital, Grenoble, France

[4]Private practice sleep and respiratory disease centre, Valence, France

[5]Private practice sleep and respiratory disease centre, Nouvelle Clinique Bel Air, Bordeaux, France

[6]Observatoire Sommeil de la Fédération de Pneumologie, France. JL Pepin est the nominated consortia representative. JPepin@chu-grenoble.fr

*These two authors contributed equally.

$These two senior authors contributed equally.

Corresponding author:

Pr. Pierre PHILIP, USR CNRS SANPSY 3413 - Bordeaux University, 33000 Bordeaux, France.

Phone: +33 (5) 57 82 01 72. Fax: +33 (5) 56 79 48 06. Email: pierre.philip@u-bordeaux.fr

QUESTIONNAIRE DU SOMMEIL :

Les informations recueillies dans ce questionnaire sont nécessaires pour la consultation avec le médecin, merci de le compléter
(Ne sont à compléter que les parties non grisées, les parties grisées seront remplies par le médecin).

IDENTIFICATION DU PATIENT:

Nom : …………………… Prénom : …………….. Date de naissance : ………….. Sexe : H ❑ F ❑

Adresse complète : ………………………………………………………………………………….

Tél : ……………………………….. Tél. portable : ……………………………………

Ville de naissance : ………………… Caractéristique de la profession : Travail posté ❑

Profession : …………………………. Métier du transport ❑

Médecin traitant : ……………..…………. Métier de la sécurité ❑

Autre ❑

ANTECEDENTS PERSONNELS : (Merci de préciser **la date de diagnostic**)

Cardiovasculaires :	Pulmonaires :
❑ Hypertension artérielle<br>❑ Hypertension artérielle pulmonaire<br>❑ Infarctus du myocarde<br>❑ Insuffisance cardiaque<br>❑ Accident vasculaire cérébral **ou** accident ischémique transitoire<br>❑ Trouble du rythme cardiaque<br>❑ Artériopathie des membres inférieurs<br>❑ Hypertrophie ventriculaire gauche<br>❑ Maladie valvulaire<br>❑ Insuffisance coronaire<br>❑ Autre : ___________________________________	❑ Maladie pulmonaire restrictive<br>❑ Insuffisance respiratoire<br>❑ BPCO<br>❑ Asthme<br>❑ Rhinite<br>❑ Syndrome d'Apnées du Sommeil<br>❑ Traitement SAS en cours<br>❑ Allergie (s) : _______________________________<br>❑ Autre : ____________________________________
**Métaboliques :**	**Autres :**
❑ Diabète type II<br>❑ Diabète type I<br>❑ Hypercholestérolémie<br>❑ Hypertriglycéridémie<br>❑ Hyperuricémie<br>❑ Insuffisance rénale<br>❑ Autre : ___________________________________	❑ Maladie neurologique<br>❑ Maladie psychiatrique<br>❑ Maladie gastro-intestinale<br>❑ Reflux gastro-oesophagien<br>❑ Glaucome<br>❑ Pathologie maligne<br>❑ Dysthyroïdie

	❑ Dépression<br>❑ Autre : ______________________

FACTEURS DE RISQUE

- Tabac

Tabagisme actuel : NSP ❑ Non ❑ Oui ❑ **Si oui, nb cig/j :** ☐

Tabagisme ancien : NSP ❑ Non ❑ Oui ❑ **Si oui, nb cig/j :** ☐

↳ Tabagisme : âge de début : |___|___| ans âge de fin : |___|___| ans

»»»»»» Consommation moyenne : |___|___| cigarettes en paquets-années

- Alcool : ❑ Oui **Si oui, nb verre/j :** ☐
  ❑ Non
  ❑ NSP

- Exercice physique : Non ❑ Oui ❑ NSP ❑
  Exercice physique description ……………………………………………
  Nombre de minutes par semaine ………………. min

TRAITEMENTS EN COURS :

Nom commercial	Dose journalière	Indication	Date de début

ANTECEDENTS FAMILIAUX :

Syndrome d'apnées du Sommeil : Non ❑ Oui ❑ NSP ❑
Dysmorphie faciale : Non ❑ Oui ❑ NSP ❑
Cardiovasculaire : Non ❑ Oui ❑ NSP ❑

CONDUITE :

Permis de conduire : Non ❑ Oui ❑ NSP ❑ Classe : ❑ A ❑ B ❑ C ❑ D ❑ E
Nombre de km/an : …………………...... km Nombre d'années de conduite : ……… ans

Somnolence pendant la conduite **0 1 2 3 4 5 6 7 8 9 10**

Très faible ***Très gênant***

Accident de somnolence Non ❑ Oui ❑ NSP ❑
Presque accident de somnolence Non ❑ Oui ❑ NSP ❑

CONSULTATION SOMMEIL :

MOTIF DE LA CONSULTATION :

❑ Asthénie (fatigue) ❑ Ronflements ❑ Suspicion de SAS
❑ Somnolence diurne ❑ Autre : ___________________________ ❑ NSP

HABITUDES DE SOMMEIL :

- A quelle heure vous **couchez**-vous habituellement ? …………………….
- A quelle heure vous **réveillez**-vous le matin habituellement ? …………….
- Combien de temps mettez-vous à vous **endormir** ? ……………………. minutes
- **Durée moyenne de sommeil** par nuit : …………………….

EVALUATION CLINIQUE DES TROUBLES DU SOMMEIL :

1) Arrêts respiratoires constatés par l'entourage : ❑ Oui ❑ Non ❑ NSP

Fréquence apnée : ❑ Presque tous les jours ❑ 3 à 4 fois par semaine
❑ 1 à 2 fois par semaine ❑ 1 à 2 fois par mois
❑ Jamais ou presque jamais

Nycturie (uriner plusieurs fois la nuit) : ❑ Oui ❑ Non ❑ NSP
Si oui, fréquence : …………………

Cataplexie : ❑ Oui ❑ Non ❑ NSP

Hallucinations visuelles ou auditives : ❑ Oui ❑ Non ❑ NSP

Paralysie du sommeil : ❑ Oui ❑ Non ❑ NSP

2) Ronflements : 0 1 2 3 4 5 6 7 8 9 10

Très faible *Très gênant*

Intensité des ronflements : ❑ Légèrement plus bruyant que votre respiration
❑ Aussi bruyant que votre voix lorsque vous parlez
❑ Plus bruyant que votre voix lorsque vous parlez
❑ Très bruyant, on vous entend dans les chambres voisines

Fréquence des ronflements : ❑ Presque tous les jours
❑ 3 à 4 fois par semaine
❑ 1 à 2 fois par semaine
❑ 1 à 2 fois par mois
❑ Jamais ou presque jamais

Ronflements dérangeants : ❑ Oui ❑ Non ❑ NSP

Fréquence endormissement véhicule : ❑ Presque tous les jours
❑ 3 à 4 fois par semaine
❑ 1 à 2 fois par semaine

- ❑ 1 à 2 fois par mois
- ❑ Jamais ou presque jamais

Envie de dormir durant la journée (somnolence diurne) :

0 1 2 3 4 5 6 7 8 9 10

Très faible — *Très gênante*

Fatigue pendant la journée :

- ❑ Presque tous les jours
- ❑ 3 à 4 fois par semaine
- ❑ 1 à 2 fois par semaine
- ❑ 1 à 2 fois par mois
- ❑ Jamais ou presque jamais

Fatigue matinale : 0 1 2 3 4 5 6 7 8 9 10

Très faible — *Très gênante*

Cette fatigue est-elle ? :

- ❑ Presque tous les jours
- ❑ 3 à 4 fois par semaine
- ❑ 1 à 2 fois par semaine
- ❑ 1 à 2 fois par mois
- ❑ Jamais ou presque jamais

Céphalées matinales : 0 1 2 3 4 5 6 7 8 9 10

Très faibles — *Très gênants*

Trouble de la libido : 0 1 2 3 4 5 6 7 8 9 10

Très faible — *Très gênant*

Trouble de l'érection : 0 1 2 3 4 5 6 7 8 9 10

Très faible — *Très gênant*

Trouble de la mémoire : 0 1 2 3 4 5 6 7 8 9 10

Très faible — *Très gênant*

Transpiration nocturne : 0 1 2 3 4 5 6 7 8 9 10

Très faible — *Très gênante*

Dyspnée : 0 1 2 3 4 5 6 7 8 9 10

Très faible — *Très gênant*

MRC :	❑ 0	❑ 1	❑ 2	❑ 3	❑ 4	(NA si dyspnée = 0)
NYHA :	❑ I	❑ II	❑ III	❑ IV		(NA si dyspnée = 0)

Etat de santé : 0 1 2 3 4 5 6 7 8 9 10

Décès — *Echelle patient* — *Parfaite santé*

ECHELLE DE SOMNOLENCE D'EPWORTH :

>> Dans les 8 circonstances suivantes, avez-vous un risque de vous endormir dans la journée ?

0<br>Ne somnolerait jamais	1<br>Faible chance de s'endormir	2<br>Chance moyenne de s'endormir	3<br>Forte chance de s'endormir

1) Assis en train de lire 0 1 2 3
2) En train de regarder la télévision 0 1 2 3
3) Assis, inactif, dans un endroit public (cinéma, théâtre, réunion) 0 1 2 3
4) Comme passager dans une voiture roulant sans arrêt pendant 1 heure 0 1 2 3
5) Allongé l'après-midi pour se reposer lorsque les circonstances le permettent 0 1 2 3
6) Assis en train de parler à quelqu'un 0 1 2 3
7) Assis calmement après un repas sans alcool 0 1 2 3
8) Dans une auto immobilisée quelques minutes dans un encombrement 0 1 2 3

Score =

ECHELLE DE FATIGUE DE PICHOT :

>> Entourez le nombre qui correspond le mieux à votre état durant la semaine dernière et jusqu'à ce jour :

0<br>Pas du tout	1<br>Un petit peu	2<br>Moyennement	3<br>Beaucoup	4<br>Extrêmement

1) Je manque d'énergie 0 1 2 3 4
2) Tout demande effort 0 1 2 3 4
3) Je me sens faible à certains endroits du corps 0 1 2 3 4
4) J'ai les bras ou les jambes lourdes 0 1 2 3 4
5) Je me sens fatigué sans raison 0 1 2 3 4
6) J'ai envie de m'allonger pour me reposer 0 1 2 3 4
7) J'ai du mal à me concentrer 0 1 2 3 4
8) Je me sens fatigué, lourd, raide 0 1 2 3 4

Score =

ECHELLE DE DEPRESSION :

>> Entourez la proposition qui correspond le mieux à votre état durant la semaine dernière et jusqu'à ce jour :

1) J'ai du mal à me débarrasser des mauvaises pensées qui me passent par la tête Vrai Faux
2) Je suis sans énergie Vrai Faux
3) J'aime moins qu'avant faire les choses qui me plaisent ou m'intéressent Vrai Faux
4) Je suis déçu et dégouté par moi-même Vrai Faux
5) Je me sens bloqué ou empêché devant la moindre chose à faire Vrai Faux
6) En ce moment je suis moins heureux que la plupart des gens Vrai Faux
7) J'ai le cafard Vrai Faux
8) Je suis obligé de me forcer pour faire quoi que ce soit Vrai Faux
9) J'ai l'esprit moins clair que d'habitude Vrai Faux
10) Je suis incapable de me décider aussi facilement que de coutume Vrai Faux
11) En ce moment, je suis triste Vrai Faux
12) J'ai du mal à faire les choses que j'avais l'habitude de faire Vrai Faux

13) En ce moment, ma vie me semble vide. Vrai Faux

Score (nombre de « vrai ») =

SYNDROME DES JAMBES SANS REPOS :

Appelé aussi « Impatiences », le syndrome des jambes sans repos est un syndrome neurologique sensitivo-moteur. Il se manifeste par des sensations désagréables, parfois douloureuses, ressenties dans les pieds, dans les jambes et parfois dans les bras.
Ces sensations sont décrites comme des fourmillements, picotements, brûlures, contractures, secousses, torsions, décharges électriques, qui surviennent exclusivement au repos, principalement en position assise ou couchée, le soir et la nuit, à l'endormissement ou au cours du sommeil

>> Entourez la réponse qui correspond le mieux à vos sensations :

1) Un besoin impérieux de bouger les jambes, habituellement accompagné ou causé par des sensations inconfortables et désagréables dans les jambes (les membres supérieurs ou les autres parties du corps peuvent parfois être affectés en plus des jambes) :

Oui Non

2) Le besoin impérieux de bouger les jambes ou les sensations désagréables apparaissent ou s'aggravent lors des périodes de repos ou d'inactivité, particulièrement en position allongée ou assise :

Oui Non

3) Le besoin impérieux de bouger les jambes ou les sensations désagréables sont partiellement ou totalement soulagées par les mouvements, tels que la marche ou l'étirement, au moins aussi longtemps que dure l'activité :

Oui Non

4) Le besoin impérieux de bouger les jambes ou les sensations désagréables sont plus marqués le soir ou la nuit que pendant la journée ou ne surviennent qu'en soirée ou la nuit :

Oui Non

Nombre de réponses OUI =

EXPLORATION DU SOMMEIL :

Renseigné par votre médecin, selon les examens réalisés (polysomnographie, polygraphie, …)

EXPLORATION FONCTIONNELLE RESPIRATOIRE :

Renseigné par votre médecin, selon les examens réalisés (spirométrie, pléthysmographie, gaz du sang, …)

EXPLORATION CARDIAQUE :

Renseigné par votre médecin, selon les examens réalisés (échographie cardiaque, doppler, …)

STRATEGIES THERAPEUTIQUES PROPOSEES :

Renseignées par votre médecin, selon sa décision.

LES EVENEMENTS MEDICAUX DE SUIVI :

Renseignés par votre médecin, selon votre déclaration.

Date visite n°1 : ______ Visite effectuée par : ______

Patient adressé par : ❑ Médecin traitant
❑ Médecin spécialiste : _______________
❑ Patient lui-même
❑ Autre professionnel de santé

◙ **DONNEES ANTHROPOMETRIQUES :**

**Taille**		cm
**Poids**		kg
**Périmètre cervical**		cm
**Périmètre abdominal**		cm
**Tour de hanches**		cm
**Tour de cuisses**		cm
**Tour de mollet**		cm

◙ **SIGNES VITAUX :**

**P.A.Systolique**		mmHg
**P.A.Diastolique**		mmHg
**Fréquence cardiaque**		bpm
**Fréquence respiratoire**		/min

◙ **BILAN BIOLOGIQUE :**

Date : __ / __ / __

**Glycémie**		mmol/L
**Cholestérol total**		mmol/L
**Cholestérol HDL**		mmol/L
**Cholestérol LDL**		mmol/L
**Triglycérides**		mmol/L
**CRPus**		mg/L
**Créatinine**		µmol/L
**HBA1c**		%
**Microalbuminurie**		mg/L
**Créatinine urinaire**		mmol/L
**HCO_3^-**		mmol/L

Profil maxillofacial semble être un facteur dans le SAS : ❑ Oui ❑ Non ❑ NSP

Classe de Mallampati : ❑ I ❑ II ❑ III ❑ IV ❑ NSP

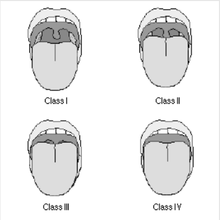

<u>CLINICAL GLOBAL IMPRESSION – Severity</u> :

1 ❑ Normal, not at all ill
2 ❑ Borderline mentally ill
3 ❑ Midly ill
4 ❑ Moderately ill
5 ❑ Markedly ill
6 ❑ Severely ill
7 ❑ Among the most extremely ill patients

➢ Annexe à ne remplir que si le patient est <u>insuffisant cardiaque</u>

QUESTIONNAIRE DU MINNESOTA :

Self-reported sleepiness and not the apnoea hypopnoea index is the best predictor of sleepiness-related accidents in obstructive sleep apnoea

P Philip[1*], S Bailly[2,3*], M Benmerad[2,3], JA Micoulaud-Franchi[1], Y Grillet[4,6], M Sapène[5,6], I Jullian-Desayes[2,3], M Joyeux-Faure[2,3], R Tamisier[2,3$], JL Pépin[2,3,6$]

[1]SANPSY-USR 3413, SANPSY-CNRS, FRANCE,

[2]HP2 laboratory, INSERM U1042, Grenoble Alpes University, Grenoble, France

[3]EFCR Laboratory, Pole Thorax et Vaisseaux, Grenoble Alps University Hospital, Grenoble, France

[4]Private practice sleep and respiratory disease centre, Valence, France

[5]Private practice sleep and respiratory disease centre, Nouvelle Clinique Bel Air, Bordeaux, France

[6]OSFP : *Observatoire Sommeil de la Fédération de Pneumologie*, France. JL Pepin is the nominated consortia representative. JPepin@chu-grenoble.fr

*These two authors contributed equally.

$These two senior authors contributed equally.

Corresponding author:

Pr. Pierre PHILIP, USR CNRS SANPSY 3413 - Bordeaux University, 33000 Bordeaux, France.

Phone: +33 (5) 57 82 01 72. Fax: +33 (5) 56 79 48 06. Email: pierre.philip@u-bordeaux.fr

STROBE Statement—Checklist of items that should be included in reports of ***cohort studies***

	Item No	Recommendation	Page
**Title and abstract**	1	(*a*) Indicate the study's design with a commonly used term in the title or the abstract	1 & 2
		(*b*) Provide in the abstract an informative and balanced summary of what was done and what was found	1 & 2
**Introduction**			
Background/rationale	2	Explain the scientific background and rationale for the investigation being reported	3
Objectives	3	State specific objectives, including any prespecified hypotheses	4
**Methods**			
Study design	4	Present key elements of study design early in the paper	5
Setting	5	Describe the setting, locations, and relevant dates, including periods of recruitment, exposure, follow-up, and data collection	5
Participants	6	(*a*) Give the eligibility criteria, and the sources and methods of selection of participants. Describe methods of follow-up	5
		(*b*) For matched studies, give matching criteria and number of exposed and unexposed	NA
Variables	7	Clearly define all outcomes, exposures, predictors, potential confounders, and effect modifiers. Give diagnostic criteria, if applicable	5
Data sources/ measurement	8*	For each variable of interest, give sources of data and details of methods of assessment (measurement). Describe comparability of assessment methods if there is more than one group	5
Bias	9	Describe any efforts to address potential sources of bias	5
Study size	10	Explain how the study size was arrived at	5
Quantitative variables	11	Explain how quantitative variables were handled in the analyses. If applicable, describe which groupings were chosen and why	6
Statistical methods	12	(*a*) Describe all statistical methods, including those used to control for confounding	6
		(*b*) Describe any methods used to examine subgroups and interactions	6
		(*c*) Explain how missing data were addressed	6
		(*d*) If applicable, explain how loss to follow-up was addressed	6
		(*e*) Describe any sensitivity analyses	6
**Results**			
Participants	13*	(a) Report numbers of individuals at each stage of study—eg numbers potentially eligible, examined for eligibility, confirmed eligible, included in the study, completing follow-up, and analysed	7
		(b) Give reasons for non-participation at each stage	7
		(c) Consider use of a flow diagram	7
Descriptive data	14*	(a) Give characteristics of study participants (eg demographic, clinical, social) and information on exposures and potential confounders	7
		(b) Indicate number of participants with missing data for each variable of interest	7
		(c) Summarise follow-up time (eg, average and total amount)	7
Outcome data	15*	Report numbers of outcome events or summary measures over time	7
Main results	16	(*a*) Give unadjusted estimates and, if applicable, confounder-adjusted	7

		estimates and their precision (eg, 95% confidence interval). Make clear which confounders were adjusted for and why they were included	
		(*b*) Report category boundaries when continuous variables were categorized	7
		(*c*) If relevant, consider translating estimates of relative risk into absolute risk for a meaningful time period	7
Other analyses	17	Report other analyses done—eg analyses of subgroups and interactions, and sensitivity analyses	7
**Discussion**			
Key results	18	Summarise key results with reference to study objectives	8
Limitations	19	Discuss limitations of the study, taking into account sources of potential bias or imprecision. Discuss both direction and magnitude of any potential bias	10
Interpretation	20	Give a cautious overall interpretation of results considering objectives, limitations, multiplicity of analyses, results from similar studies, and other relevant evidence	9
Generalisability	21	Discuss the generalisability (external validity) of the study results	8
**Other information**			
Funding	22	Give the source of funding and the role of the funders for the present study and, if applicable, for the original study on which the present article is based	5

*Give information separately for exposed and unexposed groups.

Note: An Explanation and Elaboration article discusses each checklist item and gives methodological background and published examples of transparent reporting. The STROBE checklist is best used in conjunction with this article (freely available on the Web sites of PLoS Medicine at http://www.plosmedicine.org/, Annals of Internal Medicine at http://www.annals.org/, and Epidemiology at http://www.epidem.com/). Information on the STROBE Initiative is available at http://www.strobe-statement.org.

Multivariate model with sleepiness at the wheel included

Effect	OR [95% CI]	P-value
Age (for 10 years increase)	0.95 [0.90 ; 1.00]	0.04
BMI (for 10 kg/m² increase)	0.92 [0.83 ; 1.01]	0.07
Sex (men)	1.43 [1.26 ; 1.64]	<.01
Sleepiness at the wheel	9.30 [7.91 ; 10.93]	<.01
Epworth sleepiness scale	1.22 [1.18 ; 1.27]	<.01
Nocturnal sweating	0.79 [0.70 ; 0.88]	<.01
Memory complaints	1.55 [1.35 ; 1.79]	<.01
Periodic leg movements	1.25 [1.09 ; 1.43]	<.01
AHI (10/hour)	1.00 [0.92 ; 1.09]	0.96
Profession (Senior officers, Highly qualified manager)		<.01
Farmer	1.34 [0.97 ; 1.84]	
Dealers, craftsman	1.30 [0.96 ; 1.74]	
Intermediate profession	1.09 [0.84 ; 1.42]	
Employees	1.42 [1.13 ; 1.77]	
workers	1.24 [0.92 ; 1.67]	
Retired workers	1.63 [1.29 ; 2.06]	
No profession	1.13 [0.83 ; 1.56]	
other	1.61 [1.24 ; 2.08]	
